# Global research on the utilization of population pharmacokinetic model: a bibliometric analysis from 2000 to 2024

**DOI:** 10.3389/fphar.2025.1548023

**Published:** 2025-05-12

**Authors:** Yucheng Yao, Liyuan Zhang, Dan Chen, Shiran Qin, Mingyu Meng, Qiuyan Guo

**Affiliations:** ^1^ Department of Pharmacy, Guangxi Academy of Medical Sciences and the People’s Hospital of Guangxi Zhuang Autonomous Region, Nanning, China; ^2^ School of Pharmacy, Guangxi Medical University, Nanning, China

**Keywords:** population pharmacokinetic model, bibliometrics, visualization analysis, CiteSpace, VOSviewer

## Abstract

**Objective:**

Population pharmacokinetic (PPK) model is capable of identifying the factors that influence the variability of pharmacokinetic (PK) profiles and the degree of their influence, effectively reduces unexplained variability, and demonstrates excellent predictive ability. PPK model has been successfully constructed in several populations for a variety of drugs. However, no study has yet conducted a bibliometric analysis of publications related to the PPK model. This study aims to provide a comprehensive overview of the research progress and hotspots in the field of PPK model research through bibliometric methods.

**Methods:**

A systematic search of the Web of Science database was conducted to collect articles and reviews related to the PPK model between 2000 and 2024. We then analyzed the data using Bibliometrix R package, Microsoft Office Excel, CiteSpace and VOSviewers.

**Results:**

Between 2000 and 2024, we identified a total of 6,125 papers and 128,856 citations. The average annual growth rate of the relevant publications was 10.35%, showing continued growth momentum. These research outputs are concentrated in North America, Western Europe, and East Asia, with USA leading the way with 2,340 publications and having the highest H-index (93) and total citations (54,965). Uppsala University and *British Journal of Clinical Pharmacology* are the institutions with the highest publication output and the most influential journals, respectively. Most of the funding agencies are from the USA and the subject categories for most publications are Pharmacology Pharmacy. In terms of author contributions, professor Karlsson MO is the leader in the field with 149 publications. In addition, wo found that “critically ill patients,” “tacrolimus,” “machine learning,” “external evaluation,” “polymyxin b,” “voriconazole,” “extracorporeal membrane oxygenation,” “dose optimization” and “model-informed precision dosing” are current research hotspots and future research trends.

**Conclusion:**

This study is the first comprehensive overview of the development of PPK model and research hotspots using bibliometric methods. Our findings provide researchers, especially beginners, with insights into the application area of PPK model, helping them to grasp key information in the field.

## 1 Introduction

The Population pharmacokinetic (PPK) model is an advanced quantitative analysis tool that is primarily used to evaluate typical Pharmacokinetics (PK) parameters and the between-subject variability (BSV) and within-subject variability (WSV) in the processes of drug absorption, distribution, metabolism, and excretion (ADME). It also helps in identifying and quantifying key covariates that affect population PK parameters, such as demographic characteristics and biochemical indicators (Ette and Williams, 2004). It is essentially a data-driven model that relies on inputting a certain amount of clinical or experimental data into a model and calculating that data to predict the behavioral patterns of a drug in different individuals or groups of individuals (Goutelle et al., 2022). Compared to the traditional PK approach, the PPK model has significant advantages. The traditional PK approaches typically require dense data (≥6 samples per participant), and these sample data are usually screened through strict inclusion and exclusion criteria elements to minimize BSV. This not only increases the cost of the analysis, but also causes the inconvenience to participants. In contrast, the PPK model can handle sparse data (each participant only needs to provide one to a few samples). Moreover, participants are generally closer to the real population treated with the target medication, so the PPK model can better estimate BSV while also being more cost-effective and efficient (Ette and Williams, 2004; Kiang et al., 2012). In addition, thanks to its ability to quantitatively describe the relationship between drugs, organisms and diseases, the PPK model has been widely used in a variety of practice areas such as the quantitative design of clinical trials for new drugs, the optimization of clinical dosage delivery, and the development of individual medication regimens (Kleinberg, 2003; Kiang et al., 2012).

Over the past 20 years, the field of PPK model applications has experienced sustained and measurable growth, and a large number of relevant research results have been published in numerous academic journals. However, the ensuing problem is that the rapid growth in the number of publications makes it increasingly difficult for researchers to keep abreast of the latest research advances and findings in this PPK modeling research area in the first instance. Although many reviews and meta-analyses of the PPK model have been published, most of these studies have focused on generalizing the model to specific populations or drugs (Peng et al., 2022; Li X. et al., 2024; Yang et al., 2024). There is no comprehensive analysis of PPK model application related research on development trends, research hotspots, and future directions.

Bibliometrics has received widespread attention in recent years. As a methodology that allows rapid quantitative and qualitative analysis of scientific results and research developments, bibliometrics plays a key role in assessing the quantity and quality of publications, including books and journal articles (Thompson and Walker, 2015; Tao et al., 2020; Romanelli et al., 2021). Bibliometrics, through the use of statistical and mathematical methods, is able to construct a knowledge map of a specific field of research, thereby identifying trends in the development and predicting future emerging trends within those fields (Thompson and Walker, 2015; Wang et al., 2019; Li et al., 2020). It has been shown that the visual analysis of the knowledge structure and current research hotspots of a specific field can not only clearly reveal the research progress and technological development path of the field, but also provide a theoretical foundation and research direction for future research in the field (Yang et al., 2021).

In this study, we will use bibliometric methods to conduct a comprehensive analysis of publications with PPK model from 2000 to 2024. The analysis specifically covers a number of aspects such as annual publications, countries, institutions, authors, source journals, references and author keywords, all of which will be presented through visualizations. Our aim is to sort out trends in the number of publications on PPK model applications, identify key contributors to the field (including countries, journals, institutions, and authors), as well as reveal current research hotspots and potential emerging topics that may be addressed in the future. The kind of comprehensive analysis will provide valuable insights to researchers new to the field and help them better understand the current state of application and future direction of PPK modeling.

## 2 Methods

### 2.1 Data source and search strategy

Web of Science (WoS) is one of the most comprehensive and authoritative databases of scholarly information in the world. It contains more scientific publications than any other database, covers over 12,000 high-quality journals, and provides detailed citation records. As a result, WoS is by far the most commonly used database for bibliometric research (Wu et al., 2021a; Pei et al., 2022). In our study, all scientific publication data were derived from WoS, ensuring the quality and reliability of the research materials.

We set the search strategy to TI = (“population pharmacokinetic* model*” OR “PPK model*” OR “popPK model*” OR “population PK model*” OR NONMEM OR “non-linear mixed effects model*” OR “Model-informed Precision Dosing”) OR AB = (“population pharmacokinetic* model*” OR “PPK model*” OR “popPK model*” OR “population PK model*” OR NONMEM OR “non-linear mixed effects model*” OR “Model-informed Precision Dosing”) OR AK = (“population pharmacokinetic* model*” OR “PPK model*” OR “popPK model*” OR “population PK model*” OR NONMEM OR “non-linear mixed effects model*” OR “Model-informed Precision Dosing”). We then excluded publications including meeting abstract, early access, proceeding peer, correction, letter, editorial material, expression of concern, retracted publication, book chapters, software review and non-English words of literature types from the search results. It is then exported as “full record and cited references” and saved in plain. txt format (Wu et al., 2021b). Finally, these files were imported into the CiteSpace software and then analyzed for duplicate publications.

### 2.2 Data analysis and software tools

We used Bibliometrix R package (4.3.0) (Aria and Cuccurullo, 2017) and Microsoft Office Excel to analyze and report on basic quantitative data such as the number of publications per year, the number of publications per country and per institution, the distribution of journals, the contribution of authors, and citations. We also successfully extracted the Hirsch index (H-index) (Ioannidis et al., 2019; Shen et al., 2022) of countries, institutions, journals and authors using the “Citation Report” function in the WoS database. Journal impact factor (JIF) (Garfield, 2006; Atallah Á et al., 2020) and Quartile in category (Q1, Q2, Q3 and Q4) were also captured in the 2023 Journal Citation Report.

We used VOSviewer (veision 1.6.20) (van Eck and Waltman, 2010), CtieSpace (6.4. R1) (Synnestvedt et al., 2005; Chen et al., 2014) and Origin 2024, three software programs frequently used in bibliometrics, for visualization and analysis (Wu et al., 2021c). VOSviewer is a powerful tool for extracting key parameters from scientific publications to support analyses such as co-authorship, co-citation, and co-occurrence (Yeung and Mozos, 2020; Wu et al., 2021a). It provides a variety of intuitive visualization network view options, including Basic Network Visualization, Overlay Visualization, and Density Visualization. The software is known for its easy-to-use process and the beauty of the generated graphics (van Eck and Waltman, 2010). In this study, the software was primarily used for country co-authorship analysis, institution co-authorship analysis, author co-authorship analysis, author co-citation analysis, and author keyword co-occurrence analysis.

Another widely recognized bibliometric software is CiteSpace, developed by Chen. As one of the current mainstream tools for the visual analysis of scientific literature, CiteSpace is widely used to explore the knowledge structure, distribution, and development trends in specific research fields. In our study, CiteSpace was used to conduct timeline of the reference co-citation network, a dual-map overly of scientific journals and identify the top 25 references with the strongest citation bursts. Additionally, the software Origin was used to create the visualization map of international collaboration analysis among different countries.

## 3 Results

**FIGURE 1:**
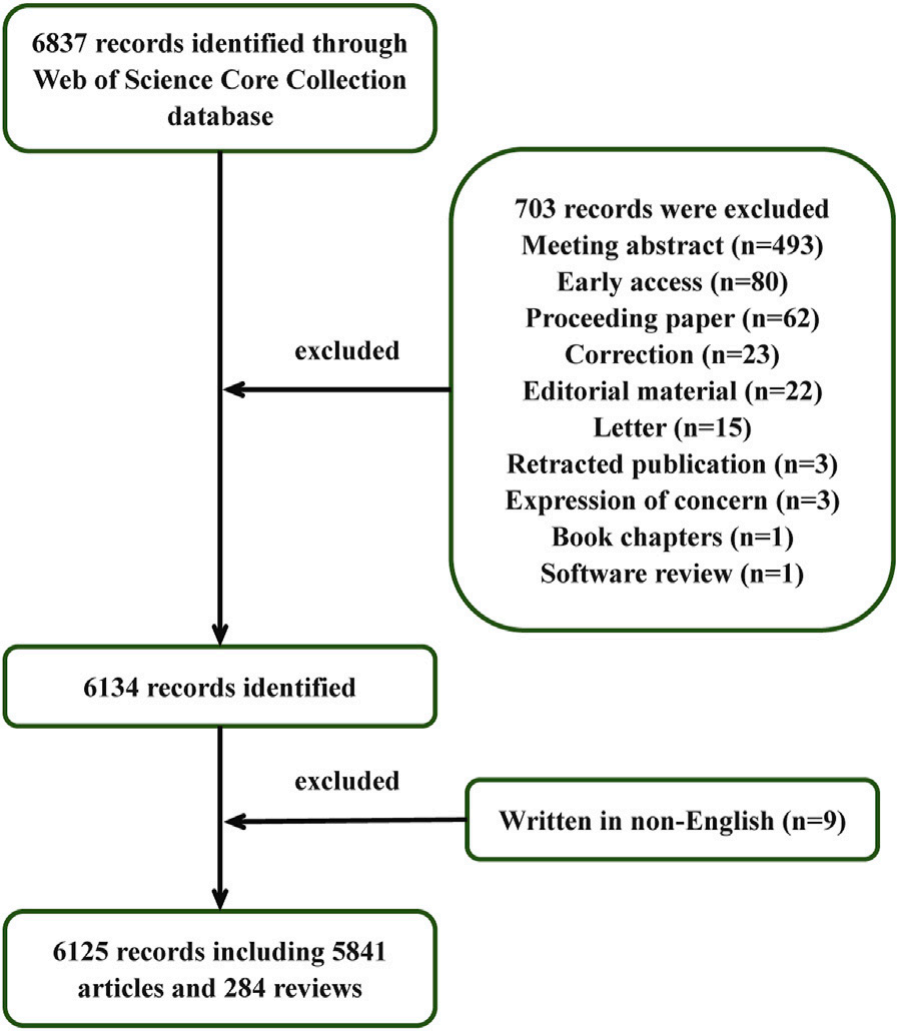
Flowchart for the data selection process.

The initial search conducted in the WoS database yielded 6,837 relevant publications. Following the implementation of exclusion criteria, 712 records were systematically removed from the analysis. Consequently, a total of 6,125 publications were retained for final examination, comprising 5,841 research articles and 284 review articles. A detailed schematic representation of the inclusion/exclusion protocol is provided in Figure 1, which outlines the complete selection workflow.

### 3.1 Global trend in publication outputs and citations

**FIGURE 2:**
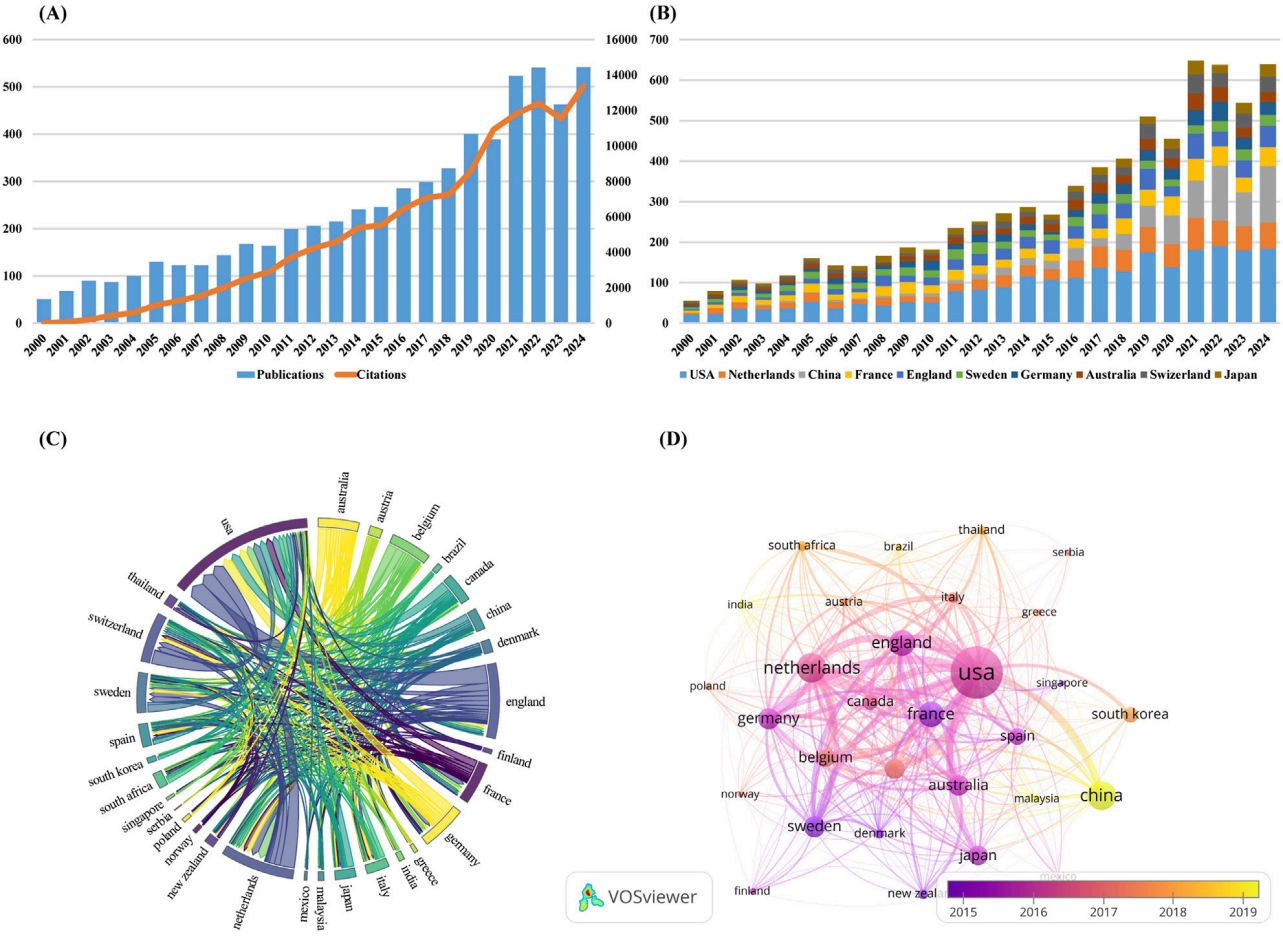
**(A)** The global trend in the annual number of PPK model-related publications and citation from 2000 to 2024; **(B)** The annual number of publications in the top 10 most productive countries; **(C)** Scientific cooperation between the top 30 countries in terms of the number of publications worldwide; **(D)** Overlay visualization map of country co-authorship analysis generated by VOSviewer software.

The number of publications over a given period of time is an important and meaningful indicator of the status and trends of the field of study over that period of time. Figure 2A shows the global trend in the annual number of PPK model-related publications and citation from 2000 to 2024. Although the number of publications fluctuates and declines at certain points in time, the decline is not significant and the overall trend is upward. The average annual growth rate was calculated on the basis of formula “[(number of documents in the last year ÷ number of documents in the first year)^1/(last year −first year)^ – 1] × 100” (Guo et al., 2020) and resulted in 10.35%. The annual publication output first exceeded 100 publications in 2004 and surpassed 200 publications by 2012. Notably, a marked acceleration in research productivity emerged from 2016 onward, with annual publications exceeding 300 by 2018. Despite transient declines observed in 2020 and 2023, scholarly output reached an unprecedented peak in 2024 (n = 542), accounting for 8.85% of the cumulative publications. As of 31 December 2024, the aggregated citation count attained 128,856 citations (102,859 excluding self-citations), with an average of 21.04 citations per publication. The annual growth rate of citations is 33.96%.

### 3.2 Analysis of national publication volume and cooperation

By systematically combing global publication data, we identified a total of 108 countries that have published corresponding academic literature in the field of PPK model research. Table 1 lists the top 10 countries with the most publications. The United States of America have the most publications (2,340, 38.20%), followed by Netherlands (814, 13.29%), the China (780, 12.73%), France (643, 10.50%), and England (640, 10.45%). The United States of America not only dominate in terms of the number of publications, but also in terms of academic impact, with the highest H-index (93) and total citations (TC) (54,965). For Average Citations per Item (ACI), Switzerland tops the list with 36.69, followed by Sweden with 35.55 and the Australia with 29.48. Figure 2B shows the annual number of publications in the top 10 most productive countries. As can be seen in the figure, the United States of America have maintained its leadership in the number of publications in this area. China shows a significant growth trend in the number of its publications from 2019. By calculating the average annual growth rate, we find that China has the highest average annual growth rate of 25.14%, followed by Netherlands (12.32%) and England (11.28%).

**TABLE 1:** Top 10 countries with the most publications.

Rank	Country	Counts	H-index	TC	ACI
1	United States of America	2,340	93	54,965	23.49
2	Netherlands	814	66	20,891	25.66
3	China	780	35	7,679	9.84
4	France	643	57	15,811	24.59
5	England	640	57	14,871	23.24
6	Sweden	456	57	16,210	35.55
7	Germany	453	53	11,827	26.11
8	Australia	418	57	12,323	29.48
9	Switzerland	401	47	9,500	36.69
10	Japan	367	38	5,983	16.30

Abbreviations: ACI, average citations per item; TC, total citations.

Figure 2C visualizes how scientific cooperation between the top 30 countries in terms of the number of publications worldwide. The line between countries represents the cooperative relationship between two countries, and the thickness of the line reflects the frequency of cooperation between countries. The United States of America collaborated most frequently with other countries, working most closely with England, the Netherlands, Switzerland, Germany, and Canada, collaborating on 279, 228, 208, 162, and 148 publications, respectively. Figure 2D is an overlay visualization map of country co-authorship analysis. Each node in the network view represents a country, and the size of the node is proportional to the number of publications. It is also marked with different colors according to the average year of the country’s emergence, with a gradual transition from dark purple to bright yellow. The dark purple color indicates countries that have started research in the field of PPK model earlier, while the bright yellow color represents countries that are newer to the research. It can be seen that China, Malaysia and India appear as bright yellow nodes in the network view, indicating that these three countries are emerging forces in research in the field of PPK modeling, and although their research activities may have begun in a later year, they have quickly established cooperative relationships with other countries.

### 3.3 Analysis of institutional output and cooperation

From the point of view of institutional contribution, about 5,428 institutions have conducted research related to the PPK model. Table 2 lists the top 10 institutions with the most publications in the PPK model application area. Uppsala University topped the list with 329 publications, followed by Leiden University and Erasmus MC with 261 and 184 publications respectively. The total number of publications from these 10 institutions represents 28.52% of the total number of publications in the entire field of study. Seven of these 10 institutions are from Europe, including five from the Netherlands and one each from Sweden and France. The remaining three institutions are from the United States of America, China and Australia. When evaluating the two metrics, H-index and ACI, we found that Uppsala University ranked first with an H-index of 54, followed by Erasmus MC and Leiden University with 47 and 45 respectively. In terms of ACI, Uppsala University came first with an ACI value of 42.82, followed by Queensland University and Leiden University with 37.42 and 29.36 respectively.

**TABLE 2:** Top 10 institutions with the most publications.

Rank	Institution, country	Counts	H-index	TC	ACI
1	Uppsala Univ, Sweden	329	54	14,087	42.82
2	Leiden Univ, Netherlands	261	45	7,662	29.36
3	Erasmus MC, Netherlands	184	47	4,126	22.42
4	Paris Cite Univ, France	167	40	4,225	25.30
5	Queensland Univ, Australia	154	44	5,762	37.42
6	Radboud Univ Nijmegen, Netherlands	140	29	2,865	20.46
6	Utrecht Univ, Netherlands	140	34	3,669	26.21
7	Fudan Univ, China	129	22	1,665	12.91
8	Certara, United States of America	123	19	1,465	11.91
9	Groningen univ, Netherlands	120	32	3,076	25.63

Abbreviations: ACI, average citations per item; TC, total citations.

**FIGURE 3:**
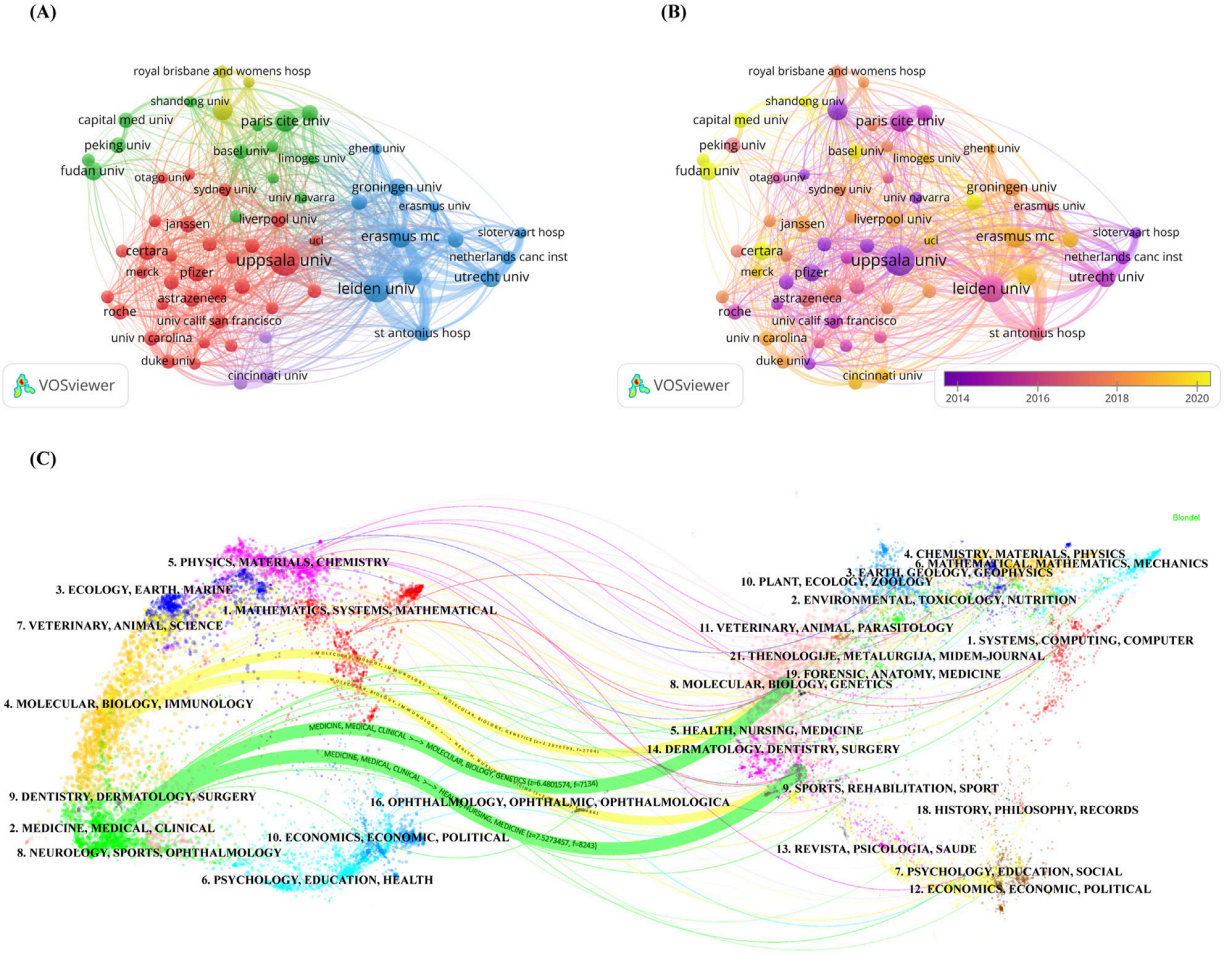
**(A)** Network visualization map of institution co-authorship analysis generated by VOSviewer software; **(B)** Overlay visualization map of institution co-authorship analysis generated by VOSviewer software; **(C)** The dual-map overlay of academic journals generated by CiteSpace software.

In addition, in order to analyze in depth the cooperation between institutions, we conducted an institution co-authorship analysis using the Vosviewer software. As shown in Figure 3A, the figure presents a visualization of institutions with at least 50 publications. There are a total of 63 institutions in the network diagram and they are classified into different clusters based on the strength of cooperation between them. Nodes of the same color indicate that they belong to the same cluster, and there are five clusters in the network graph, with the red cluster having the highest number of institutions at 31. Figure 3B shows the Overlay visualization map of institution co-authorship analysis, from which nodes marked in dark purple can be identified, such as Uppsala University, Queensland University, Pfizer and Suny Buffalo institutions’ researchers were early participants in research related to the PPK model. In contrast, researchers at institutions represented by nodes assigned a bright yellow color, such as Capital Medicine University, Fudan University, Shandong University and Certara, are likely to be more recent participants in studies related to the PPK model.

### 3.4 Analysis of most funding agencies

Supplementary Figure S1 represents the top 10 funding agencies in the PPK model research area, with the United States Department of Health Human Services ranking first with 561 studies funded. Of these 10 agencies, four are from the United States of America, one each from China, Australia, and England, and the rest are pharmaceutical companies.

### 3.5 Analysis of article output and impact of journals

In terms of publication journals, about 595 journals published studies related to PPK model. Table 3 lists the top 10 journals with the most publications. The *British Journal of Clinical Pharmacology* ranked first with 459 publications, followed by *Antimicrobial Agents and Chemotherapy* and *Clinical Pharmacokinetics* with 430 and 415 publications respectively. For H-index and ACI, *Antimicrobial Agents and Chemotherapy* ranked first with 59 and 32.06 respectively. It was followed by *Clinical Pharmacokinetics* and *British Journal of Clinical Pharmacology* with H-index and ACI of 57 and 27.04 and 51 and 25.40 respectively. Notably, *Clinical Pharmacokinetics* had the highest JIF of 4.6 in 2023. In terms of journal ratings, four journals were categorized as Q1 while among the remaining journals categorized as Q2, Q3 and Q2/Q3 there were 2, 3 and 1 respectively. For the geographical distribution of journals, the United States of America lead the way with five journals, followed by England with two journals. New Zealand, Switzerland and Germany contributed 1 journal each.

**TABLE 3:** Top 10 journals with the most publications.

Rank	Journal	Counts	H-index	JIF(2023)	JRC (2023)	Country	TC	ACI
1	British Journal of Clinical Pharmacology	459	51	3.1	Q2	England	11,657	25.40
2	Antimicrobial Agents and Chemotherapy	430	59	4.1	Q1	United States of America	13,785	32.06
3	Clinical Pharmacokinetics	415	57	4.6	Q1	New Zealand	11,221	27.04
4	Journal of Clinical Pharmacology	395	40	2.4	Q3	United States of America	7,056	17.86
5	Journal of Pharmacokinetics and Pharmacodynamics	240	35	2.2	Q3	United States of America	5,523	23.01
6	Therapeutic Drug Monitoring	221	35	2.8	Q2	United States of America	4,616	20.89
7	European Journal of Clinical Pharmacology	198	35	2.4	Q3	Germany	4,351	21.97
8	Journal of Antimicrobial Chemotherapy	196	36	3.9	Q1	England	4,460	22.76
9	Cancer Chemotherapy and Pharmacology	166	33	2.7	Q2/Q3	United States of America	4,020	24.22
10	Frontiers in Pharmacology	156	16	4.4	Q1	Switzerland	1,164	7.46

Abbreviations: ACI, average citations per item; TC, total citations.

Figure 3C shows the dual-map overlay of academic journals, which clearly depicts the distribution of topics in journals involved in PPK model research. After importing the dataset into the atlas, citation trajectories are produced, and they are visualized as colored paths in the figure (Chen and Leydesdorff, 2014; Wang et al., 2024a). This approach gives us a clear picture of the way knowledge flows between different fields of study. The mapping shows the following four core citation paths, with the green path indicating that most articles published in Medicine/Medical/Clinical journals may tend to cite articles published in Molecular/Biology/Genetics and Health/Nursing/Medicine journals. Yellow paths indicate that most articles published in Molecular/Biology/Immunology may tend to cite articles published in Molecular/Biology/Genetics and Health/Nursing/Medicine journals.

### 3.6 Analysis of the subject categories analysis

In the WoS database, every article is categorized into at least one subject category. Supplementary Figure S2 provides a visual overview showing the top 10 subject categories in terms of number of publications. We can see that the Pharmacology Pharmacy subject category leads the way with 4,897 publications. It is followed by Microbiology and Infectious Diseases with 785 and 498 publications respectively. This result indicates that PPK model is mainly applied to the research area of Pharmacology Pharmacy.

### 3.7 Analysis of author’s article output, cooperation and co-citation

The number of publications by authors is an important indicator of how active they are in the academic field and how much they contribute. In the study, more than 25,244 authors contributed 6,125 scholarly articles. Table 4 lists the top 10 authors with the most publications. Among them, Karlsson MO topped the list with 149 publications, with an H-index of 32 and an ACI of 42.23. In second place was Knibbe CAJ with 96 publications with an H-index and ACI of 35 and 35.24 respectively. Huitema ADR was ranked third with 93 publications and its H-index and ACI were 28 and 26.70 respectively. We find that European scholars dominate the list, with seven authors on the list, five from the Netherlands, one from Sweden and one from Belgium. Of the remaining three authors, they are from China, Australia and the United States of America.

**TABLE 4:** Top 10 authors with the most publications.

Rank	Author	CounAbbreviations: ts	H-index	TC	ACI	Institution and country
1	Karlsson MO	149	32	6,293	42.23	Uppsala University and Sweden
2	Knibbe CAJ	96	35	3,383	35.24	Leiden University and Netherlands
3	Huitema ADR	93	28	2,483	26.70	Utrecht University and Netherlands
4	Mathot RAA	85	23	1,803	21.21	Amsterdam University and Netherland
5	Beijnen JH	80	28	2,306	28.83	Netherlands Cancer Institute and Netherlands
6	Zhao Wei	68	21	1,401	20.60	Shandong University and China
7	Danhof M	66	32	2,300	34.85	Leiden University and Netherlands
8	Allegaert K	64	26	1,962	30.66	Leuven University and Belgium
9	Roberts JA	59	25	2,234	37.86	Queensland University and Australia
10	Vinks AA	55	21	1,565	28.45	Cincinnati University and United States of America

Abbreviations: ACI, average citations per item; TC, total citations.

**FIGURE 4:**
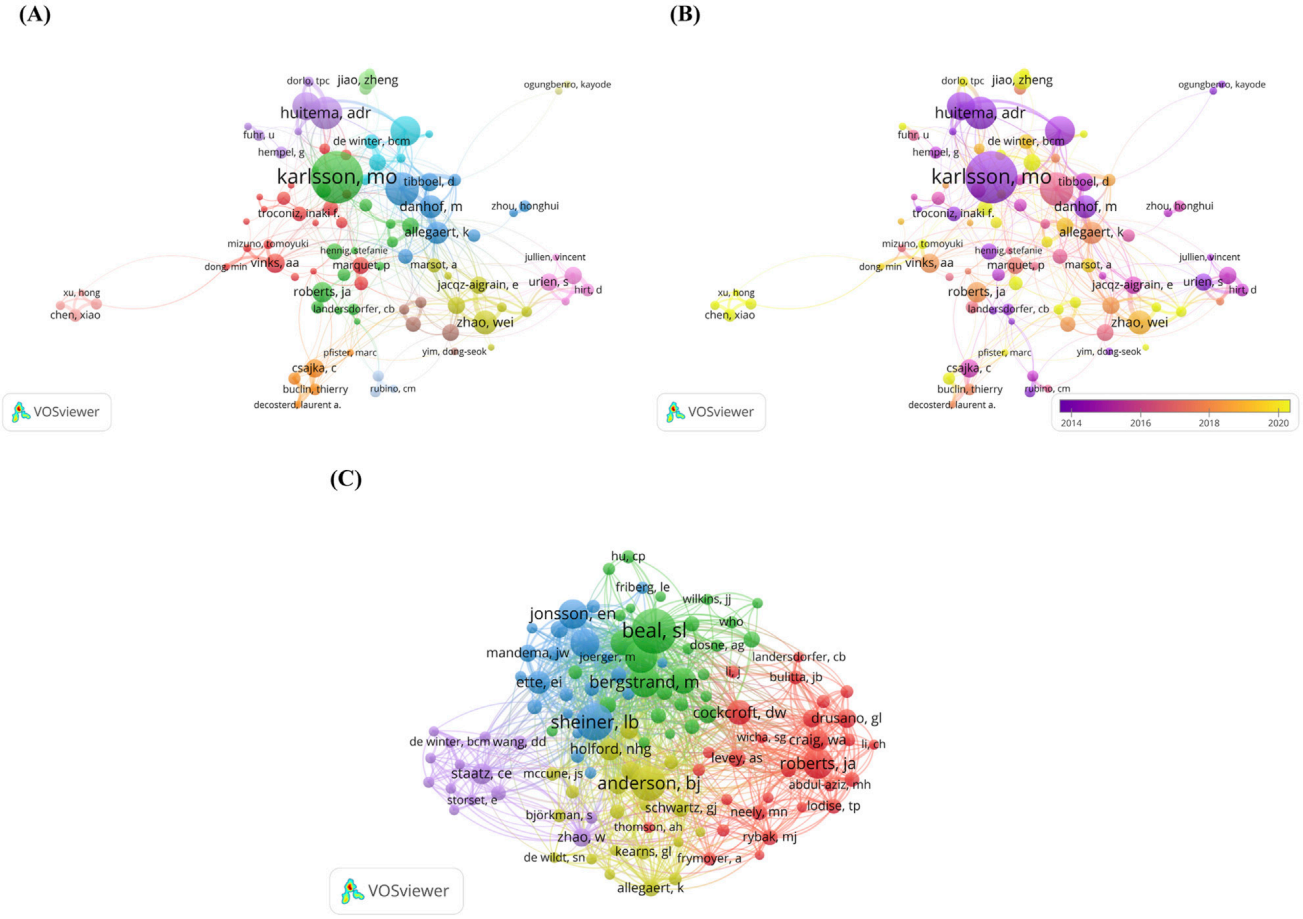
**(A)** Netwrok visualization map of author co-authorship analysis generated by VOSviewer software; **(B)** Overlay visualization map of author co-authorship analysis generated by VOSviewer software; **(C)** Network visualization map of author co-cotation analysis generated by VOSviewer software.

Figure 4A shows the network visualization map of author co-authorship analysis. There are 93 nodes in the network graph, which are divided into 13 clusters including green cluster dominated by Karlsson MO, blue cluster dominated by Knibbe, CAJ and Allegaert K, purple cluster dominated by Huitema ADR and yellow cluster dominated by Zhao Wei, *etc.* The authors contained in each cluster collaborate more frequently with each other. Figure 4B shows the Overlay visualization map of author co-authorship analysis. To improve the readability and clarity of the network map, we only show authors with at least 20 publications. The entire network graph has 93 nodes, where the authors represented by the dark purple nodes, such as Beijnen JH, Karlsson MO, Urien S and Danhof M are the earlier participants who carried out the research related to the PPK model. Whereas the authors represented by the bright yellow nodes, such as Jiao Zheng, Chen Xiao and Xu Hong may have been later participants in the PPK model study. In addition, we performed authors co-citation analysis. When two different authors, journals, or documents appeared in the reference list of a third document at the same time, it indicated that a co-citation relationship was established between them (González-Alcaide et al., 2016). Figure 4C shows network visualization map of author co-citation analysis, the visualization network shows 115 authors who received at least 100 citations. These authors were categorized into five clusters based on co-citation relationships. Among these authors, Beal SL ranked first with 1,611 high citations followed by Sheiner LB, Anderson BJ, Lindbom L and Bergstrand M with 1,119, 998, 883 and 772 citations respectively.

### 3.8 Analysis of highly-cited publications

Table 5 lists the top 10 cited publications, which were published between 2000 and 2011, of which nine were original articles and one was a meta-analysis. Each article had more than 200 citations, with the two studies published by Lindbom L et al., in 2005 (Lindbom et al., 2005) and 2004 (Lindbom et al., 2004) ranking first and second in citations, specifically 940 and 603 citations, respectively. This was followed by a study by Garonzik et al. (2011) published in 2011 with 587 citations. It is worth noting that the number of citations an article receives typically increases over time, so studies published earlier may receive higher citations due to longer dissemination times. To more accurately assess the scholarly impact of each article, we further analyzed the average annual citations of publications (Pei et al., 2022). As can be seen from Table 5, the study by Lindbom L et al. published in 2005 Lindbom et al. (2005) not only ranked first in terms of citations, but its average annual citations were also the highest in the list with 49.47 citations. It was followed by the studies of Garonzik et al. (2011) and Savic and Karlsson (2009) with 45.15 and 31.33 citations, respectively. In summary, both in terms of total citations and average annual citations reflect the significant position and notable contribution of the study published by Lindbom L et al., in 2005 to the PPK model.

**TABLE 5:** The top 10 cited publications.

Title	Frist author	Journal	Year	TC	TC per year
PsN-Toolkit - A collection of computer intensive statistical methods for non-linear mixed effect modeling using NONMEM	Lindbom L	Computer Methods and Programs in Biomedicine	2005	940	49.47
Perls-speaks-NONMEM (PsN) - a Perl module for NONMEM related programming	Lindbom L	Computer Methods and Programs in Biomedicine	2004	603	30.15
Population Pharmacokinetics of Colistin Methanesulfonate and Formed Colistin in Critically Ill Patients from a Multicenter Study Provide Dosing Suggestions for Various Categories of Patients	Garonzik SM	Antimicrobial Agents and Chemotherapy	2011	587	45.15
Importance of Shrinkage in Empirical Bayes Estimates for Diagnostics: Problems and Solutions	Savic RM	AAPS Journal	2009	470	31.33
Implementation of a transit compartment model for describing drug absorption in pharmacokinetic studies	Savic RM	Journal of Pharmacokinetics and Pharmacodynamics	2007	402	23.65
Population Pharmacokinetic Analysis of Colistin Methanesulfonate and Colistin after Intravenous Administration in Critically Ill Patients with Infections Caused by Gram-Negative Bacteria	Plachouras D	Antimicrobial Agents and Chemotherapy	2009	381	25.40
Computing normalised prediction distribution errors to evaluate nonlinear mixed-effect models: The npde add-on package for R	Comets E	Computer Methods and Programs in Biomedicine	2008	375	23.44
Relationship between exposure to sunitinib and efficacy and tolerability endpoints in patients with cancer: results of a pharmacokinetic/pharmacodynamic meta-analysis	Houk BE	Cancer Chemotherapy and Pharmacology	2010	361	25.79
A randomized comparison of native *Escherichia coli* asparaginase and polyethylene glycol conjugated asparaginase for treatment of children with newly diagnosed standard-risk acute lymphoblastic leukemia: a Children’s Cancer Group study	Avramis VI	Blood	2002	353	16.05
Population pharmacokinetics of propofol: A multicenter study	Schuttler J	Anesthesiology	2000	299	11.96

Abbreviation: TC, total citations.

### 3.9 Reference co-cited and burst analysis

**FIGURE 5:**
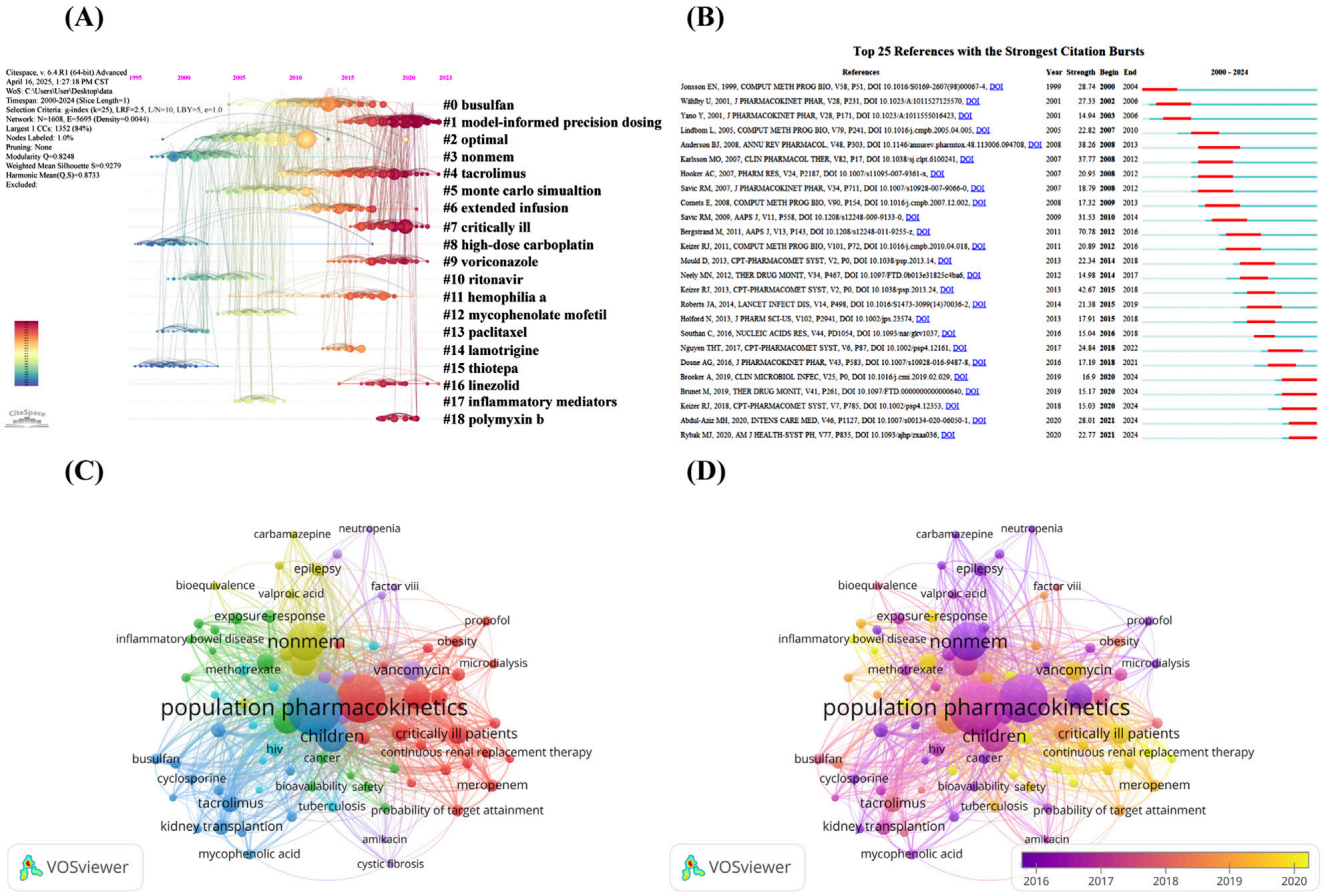
**(A)** Timeline of the reference co-citation network by Citespace software; **(B)** Visualization map of top 25 references with the strongest citation bursts by Citespace software; **(C)** Network visualization map of authors’ keywords co-occurrence analysis by VOSviewer software; **(D)** Overlay visualization map of authors’ keywords co-occurrence analysis by VOSviewer software.

High-frequency co-cited references often represent the underlying knowledge base of a particular research area (Sabe et al., 2023). Table 6 lists the top 10 cited references. Specifically, four studies explored how to assess and improve the quality and predictive performance of the PPK model (Sheiner and Beal, 1981; Beal, 2001; Savic and Karlsson, 2009; Bergstrand et al., 2011). Four studies outlined the commonly used auxiliary software used in the construction of PPK models: Pirana, PsN and Xpose (Jonsson and Karlsson, 1999; Lindbom et al., 2004; Lindbom et al., 2005; Keizer et al., 2013). Of the remaining two studies, one study explored the model used to describe the relationship between size and age and pharmacokinetic parameters in premature neonates to young adults (Anderson and Holford, 2008), and the other proposed a formula to predict creatinine clearance from serum creatinine, namely, the Cockcroft-Gault formula (Cockcroft and Gault, 1976). In addition, we produced the timeline of the reference co-citation network based on 1 year as the unit of time slice (2000–2024 as the period), as shown in Figure 5A. The modularity Q is greater than 0.3 and the weighted mean silhouette S is greater than 0.5 or 0.7 to ensure analytical reliability and accuracy (Chen et al., 2022). According to the analysis, the modularity Q is 0.8248 and the weighted mean silhouette S is 0.9279, indicating that the network diagram is reasonable. The figure shows the main 19 clusters, with high-dose carboplatin (#8) and thiotepa (#15) being the early research themes in the field, while model-informed precision dosing (#1), tacrolimus (#4), critically ill (#7), linezolid (#16) and polymyxin b (#18) are current research hotspots.

**TABLE 6:** The top 10 co-cited reference.

Title	Author	Year	Journal	Citations
Prediction-corrected visual predictive checks for diagnosing nonlinear mixed-effects models	Bergstrand M	2011	AAPS Journal	638
Prediction of creatinine clearance from serum creatinine	Cockroft DW	1976	Nephron	540
PsN-Toolkit - A collection of computer intensive statistical methods for non-linear mixed effect modeling using NONMEM	Lindbom L	2005	Computer Methods and Programs in Biomedicine	541
Xpose--an S-PLUS based population pharmacokinetic/pharmacodynamic model building aid for NONMEM	Jonsson EN	1999	Computer Methods and Programs in Biomedicine	536
Mechanism-based concepts of size and maturity in pharmacokinetics	Anderson BJ	2008	Annual Review of Pharmacology and Toxicology	384
Ways to fit a PK model with some data below the quantification limits	Beal SL	2001	Journal of Pharmacokinetics and Pharmacodynamics	368
Some suggestions for measuring predictive performance	Sheiner LB	1981	Journal of pharmacokinetics and biopharmaceutics	356
Perls-speaks-NONMEM (PsN) - a Perl module for NONMEM related programming	Lindbom L	2004	Computer Methods and Programs in Biomedicine	338
Modeling and Simulation Workbench for NONMEM: Tutorial on Pirana, PsN, and Xpose	Keizer RJ	2013	CPT Pharmacometrics Systems Pharmacology	261
Importance of shrinkage in empirical bayes estimates for diagnostics: problems and solutions	Savic RM	2009	AAPS Journal	254

Burst detection algorithm is a tool proposed by Kleinberg that can identify references or keywords with a sharp increase in the number of citations in a specific time period, which can effectively reveal the research direction and hotspots in a certain period of time (Kleinberg, 2003). In this study, we use burst detection algorithm to analyze the references of PPK model related studies. Figure 5B shows the top 25 references with the strongest citation bursts. The dark blue line in the figure represents the sustained citation time span from 2000 to 2024, while the red line indicates the duration of the citation burst, and we set the minimum burst duration to 3 years. The first co-citation burst appeared in 2001 as a result of research published by Jonsson EN et al., in 1999 (Jonsson and Karlsson, 1999). The study demonstrates in detail the auxiliary software for building PPK model: Xpose. It is noteworthy that the citation bursts of five references last until 2024, which to some extent reflects the research trends and hotspots in recent years. Two of these studies focused on individualized dosage adjustment and therapeutic drug testing of antimicrobials in critically ill patients (Abdul-Aziz et al., 2020; Rybak et al., 2020), one study discussed individualized precision dosing of tacrolimus and therapeutic drug monitoring in various types of transplant patients (Brunet et al., 2019), one study explored the model-informed precision dosing in practice with possible scientific challenges (Keizer et al., 2018), and 1 study evaluated the predictive performance of 31 established PPK models for vancomycin using Bayesian methods for external evaluation (Broeker et al., 2019). In addition, we observe that although the citation explosion period for most of the references has ended, they are still widely cited in subsequent times, suggesting that the PPK model is still widely followed among academics.

### 3.10 Keyword co-occurrence analysis

Keyword co-occurrence analysis is an effective method that can reveal the distribution of hotspots in a particular research area (Zheng et al., 2022). Figure 5C network visualization map of authors’ keywords co-occurrence analysis. We set the threshold for the number of co-occurrences to be greater than 20, and identified a total of 84 keywords after removing meaningless keywords and merging keywords with the same meaning. The size of the nodes in the network graph is directly proportional to the frequency of occurrence of the keywords, and the thickness of the connecting lines between the nodes represents the frequency of co-occurrence between the keywords. The keywords in the network diagram are categorized into six clusters, each of which is primarily related to a specific patient population and the therapeutic agents they use. The largest red clusters focus on keywords related to antimicrobials and critically ill populations, such as “critically ill patients,” “sepsis,” “meropenem,” “extracorporeal membrane oxygenation” and “polymyxin b,” among others. This is closely followed by the green cluster, which is the second largest, with keywords mainly related to autoimmune diseases and their therapeutic drugs, including “monoclonal antibody,” “inflammatory bowel disease,” “rheumatoid arthritis” and “infliximab.” The third largest blue cluster is mainly related to transplantation and covers “tacrolimus,” “kidney transplantation” and “hematopoietic stem cell transplantation” and so on. The yellow cluster comes next and is mainly related to psychiatric disorders, covering keywords such as “epilepsy,” “lamotrigine” and “valproic acid.” Finally, the purple and light blue clusters are mainly associated with hemophilia and HIV patients. Figure 5D shows the overlay visualization map of authors’ keywords co-occurrence analysis, by observing the color change of keyword nodes, we can identify the average time of occurrence of each keyword. The results reveal an evolutionary pattern of research hotspots. Specifically, between 2010 and 2014, the research hotspots appear to be “clearance,” “cyclosporine” and “propofol”; between 2015 and 2016, “bayesian forecasting” and “drug-drug interaction” received significant attention, while specific therapeutic areas such as “hiv,” “epilepsy,” “monoclonal antibody” and “methotrexate” are targeted for research; between 2017 and 2019, research focus has shifted to special populations include “children,” “critically ill patients,” “neonate” and “kidney transplantion,” while drugs such as “vancomycin” and “tacrolimus” are also receiving continued attention; notably, keywords that appear on average after 2020, such as “machine learning,” “external evaluation,” “polymyxin b,” “voriconazole,” “extracorporeal membrane oxygenation,” “dose optimization,” and “model-informed precision dosing” are likely to dominate the development process of this field in the next 5–10 years, and this trend is highly compatible with the current rapid development of precision medicine and artificial intelligence technology.

Additionally, to delve deeper into the specific populations and medications that receive the most attention in the PPK model field, we have listed the top 15 groups or drugs in terms of frequency of occurrence. Table 7 show that children are the most popular group with 494 occurrences. This was followed by “critically ill patients” and “neonate,” with 169 and 109 occurrences, respectively. In terms of specific drugs, “vancomycin” appeared most frequently, with 161 occurrences. It was followed by tacrolimus and meropenem, with 133 and 60 occurrences, respectively. These data reveal key focuses in PPK model studies, especially in terms of drug dose optimization and treatment strategies for specific populations.

**TABLE 7:** Top 15 groups/drugs with the highest frequency of occurrence.

Rank	Keywords (group)	Occurrence	Rank	Keywords (drug)	Occurrence
1	children	494	1	vancomycin	161
2	critically ill patients	169	2	tacrolimus	133
3	neonate	109	3	meropenem	60
4	KT	94	4	methotrexate	51
5	HIV	75	5	busulfan	44
6	epilepsy	69	6	cyclosporine	40
7	infants	63	7	gentamicin	38
8	obesity	53	8	mycophenolic acid	38
9	tuberculosis	49	9	infliximab	36
10	sepsis	43	10	piperacillin	35
11	HSCT	43	11	propofol	35
12	CRRT	41	12	valproic acid	33
13	LT	37	13	voriconazole	32
14	pregnancy	32	14	linezolid	32
15	rheumatoid arthritis	32	15	sirolimus	30

Abbreviations: CRRT, continuous renal replacement therapy; HSCT, hematopoietic stem cell transplantation; KT, kidney transplantation; LT, liver transplantation.

## 4 Discussion

With the rapid development of network technology, the application of PPK model in the field of medicine is getting more and more attention. In this review, we used bibliometric methods to analyze scientific publications related to PPK model applications between 2000 and 2024, and visualized the research progress and future trends in the field of PPK model applications to the academic community.

Our study shows a steady increase in the number of annual publications and citation frequency. After analyzing the main countries involved in the research, we found that the countries conducting the research were concentrated in North America, Western Europe, and East Asia. Of these, the United States of America are the highest producer, with a publication count of 2,340, far exceeding that of other countries, and it also boasts the highest H-index (93). The leading position of the United States in terms of publication volume is attributed to its advanced technologies, state-of-the-art equipment, professional researchers, sufficient research funding, and robust policy support. The analysis of funding agencies indicates that four agencies from the United States of America among the top ten funding bodies have collectively supported 1,382 research projects, accounting for 22.56% of the total. Additionally, the U.S. Food and Drug Administration (FDA) in its 2022 *Population Pharmacokinetics Guidance for Industry* encourages researchers to adopt PPK models in areas such as new drug applications (NDAs) and biologics license applications (BLAs). In addition, six of the top ten countries by publication volume are from Europe, having collectively published 3,407 papers, which accounts for more than half of the total. This highlights Europe’s significant contribution to the field of PPK model research. It is also noteworthy that the number of publications from China has increased significantly in recent years, making a substantial contribution to global research output. This trend is likely closely related to China’s rapid development in the field, increased research investment, and advancements in relevant technologies. Although China ranks second in publication volume, its ACI is 9.84, significantly lower than that of other countries. This may indicate that while the number of publications has increased, there is still a need to enhance the quality and impact of these publications. In addition to the significant impact of economic and policy factors on the growth of publication volume in this field, improvements in relevant technologies have also played a crucial role. For example, the development of modeling platforms and the advancement of model diagnostic tools. Besides the widely used and earliest developed software, NONMEM (Beal and Sheiner, 1980), several new modeling platforms, such as Monolix, Phoenix NLME, and mrgsolve, were developed around 2010. These software programs are easier to learn and use compared to NONMEM. In terms of model diagnostic tools, the introduction of methods such as the prediction-corrected visual predictive check and normalized prediction distribution error has significantly enhanced the verifiability and reliability of PPK models. Additionally, the growing emphasis on personalized medicine in clinical practice is another important factor contributing to the development of this field (Goetz and Schork, 2018). In terms of international collaboration, the United States of America have the most frequent partnerships with other countries. However, current international cooperation is mainly concentrated between developed European countries and the United States of America. Therefore, we believe that future efforts should focus on strengthening international collaboration, particularly by involving more countries, including developing nations. This will help promote the overall development of the PPK model research globally.

Regarding publishing institutions, Uppsala University stands out with 329 publications, far surpassing other institutions. In addition to its large volume of publications, the institution also maintains high research quality, with five of its studies ranking among the top 10 most-cited publications (Lindbom et al., 2004; Lindbom et al., 2005; Savic et al., 2007; Plachouras et al., 2009; Savic and Karlsson, 2009). Furthermore, two studies published by the institution in 2008 and 2009 are also noteworthy (Ahn et al., 2008; Bergstrand and Karlsson, 2009). In clinical or preclinical research, certain low-dose or rapidly eliminated drugs often result in drug concentrations below the quantification limit (BQL). If these data are not appropriately handled, it can lead to significant bias in model estimation. Therefore, these two studies validated the applicability of different BQL handling methods in complex model structures, laying the foundation for the subsequent standardization of BQL modeling practices. Additionally, we observed that although the United States of America and China rank first and third in publication volume worldwide, only one institution from each of these countries is listed among the top 10 institutions by publication count, while the Netherlands has five institutions in the top 10. This intriguing phenomenon may suggest that institutions conducting PPK model research in the Netherlands are relatively concentrated and limited in number, while research institutions in the United States of America and China are more dispersed and numerous. Regarding institutional collaboration, Figure 3A shows that although five institutions in China contributed a substantial number of publications, their collaboration with other institutions is relatively limited, with current collaborations mainly focused on institutions in the United States of America and Western Europe. This indicates that Chinese research institutions lack sufficient academic exchange with foreign institutions, and future efforts should focus on strengthening collaboration with other institutions. Enhancing cooperation can, to some extent, break down academic barriers and improve research competitiveness and article quality. In summary, both national and institutional analyses show that China is one of the most active countries in the field of PPK modeling in recent years. It is reasonable to speculate that Chinese influence in this field will continue to grow.

In terms of journal impact, the top 10 journals by publication volume account for 46.96% of the publications in this field. Based on three key metrics—publication volume, H-index, and ACI—*British Journal of Clinical Pharmacology*, *Antimicrobial Agents and Chemotherapy*, and *Clinical Pharmacokinetics* are undoubtedly the core journals in the field of PPK modeling research. We therefore recommend that researchers prioritize the submission of their research results to these journals and reasonably predict that these journals will publish more outstanding and high-quality scholarly articles in the future. It is noteworthy that the top 10 journals all originate from the United States of America and European countries. Although two East Asian countries, China and Japan, have also contributed a great deal of research in this field, Asian journals are not yet represented in the top 10 journals. This phenomenon suggests that in the future, Asian countries, especially China and Japan, should commit themselves to enhance the development and construction of international journals in order to increase their academic influence in this field.

After analyzing the authors, we found that many scholars have carried out extensive research in the field of PPK model, and different authors show significant diversity in their research interests. Among the top ten authors by publication volume, Karlsson MO’s number of publications far exceeds that of other authors. In addition to developing multiple PPK models, he has also published two influential studies that have had a profound impact on model development and evaluation. Specifically, in his 2007 study, he compared the performance of the lag time model (LAG model) and the transit compartment model (TRANSIT model), concluding that the TRANSIT model is an ideal alternative to the LAG model when the latter poorly describes drug absorption or is numerically unstable (Savic et al., 2007). In his 2009 study, he investigated the impact of individual parameter bias on overall mean shrinkage (η-shrinkage) and residual error shrinkage towards zero (ε-shrinkage) in graphical diagnostics based on Empirical Bayes Estimates (EBEs) (Savic and Karlsson, 2009). The results indicated that when η-shrinkage and ε-shrinkage exceed 20%–30%, EBEs-based diagnostic plots may fail and become misleading. Therefore, when using these diagnostic plots, shrinkage values should be reported, and if the values are too high, more robust methods such as simulation diagnostics and conditional weighted residuals (CWRES) should be used. As shown in Figure 4A, the collaboration between Huitema ADR, and Beijnen JH is particularly close, with their research primarily focusing on the pharmacokinetics of monoclonal antibodies and cancer-related drugs. Furthermore, in 2008, they published the first systematic report on the PPK model of miltefosine in leishmaniasis patients, laying a significant foundation for subsequent research in this field (Dorlo et al., 2008). As for the other authors, the collaboration between Knibbe CAJ, Allegaert K, and Danhof M is particularly close. These three professors have focused on pharmacokinetics research in three special populations: children, infants, and neonates. Among their contributions, a study involving Professor Knibbe CAJ first systematically developed a PPK model for anti-thymocyte globulin in pediatric hematopoietic stem cell transplantation (Admiraal et al., 2015). In another study, she established the first PPK model for paracetamol spanning from preterm neonates to adults, providing a theoretical foundation for age-specific dose adjustments (Wang et al., 2014). Additionally, she and Professor Allegaert K developed the first PPK model for propofol in both preterm and term neonates, clearly identifying postmenstrual age (PMA) and postnatal age (PNA) as key covariates influencing variability in clearance (Allegaert et al., 2007). In summary, these three professors have significantly advanced the individualization and precision of pediatric drug therapy. Mathot RAA has made significant contributions to the development of PPK models related to blood coagulation factors. In a study he participated in 2016, the first PPK model for factor Ⅷ in hemophilia A patients during the perioperative period was developed, and this model is expected to evolve into an important tool for clinical dose decision-making (Hazendonk et al., 2016). Roberts JA has made significant contributions to the pharmacokinetics of antibiotics in critically ill patients. In 2009, he established a PPK model for meropenem using 222 plasma concentration samples, and for the first time compared the effects of intermittent bolus dosing and continuous infusion on the subcutaneous tissue and plasma concentration-time profiles in critically ill patients receiving meropenem (Roberts et al., 2009), Additionally, he used Monte Carlo simulations to model different dosing regimens for intermittent bolus, extended infusion, and continuous infusion, evaluating the cumulative fraction of response (CFR) for meropenem against common Gram-negative pathogens in the intensive care unit. The results indicated that continuous infusion, compared to intermittent bolus dosing, maintained higher subcutaneous tissue and plasma concentrations, and for less sensitive pathogens, extended or continuous infusion dosing achieved a higher CFR than intermittent bolus dosing. In addition, regarding author co-citation analysis, it is worth mentioning the pioneers Sheiner LB and Beal SL. Sheiner LB formally introduced the concept of population analysis in 1972 and first analyzed clinically sparse data and accessed population pharmacokinetic profiles in 1977 using digoxin as an example of a nonlinear mixed-effects modeling theory (Sheiner et al., 1977). Subsequently, in 1980, Sheiner LB and Beal SL successfully developed the first population pharmacokinetic-pharmacodynamic calculation software, NONMEN. From its successful development until today, the software has undergone many improvements and upgrades, and has now become the most widely used analytical tool in the field of PPK model. The successful development of NONMEN software marks an important step from theory to practice in population pharmacokinetics.

Among the top 10 most-cited publications, five studies explored the auxiliary software needed during model development, how to improve model performance, and how to evaluate models. In the remaining studies, two established PPK model for critically ill patients. In a 2009 study by Plachouras D et al., the first PPK model describing the *in vivo* process of colistin methanesulfonate (CMS) and colistin in critically ill patients was developed (Plachouras et al., 2009). The results suggested that the standard dose of CMS during this period might lead to insufficient early exposure to colistin, and they recommended considering a loading dose regimen for dose optimization. Similarly, Garonzik SM et al. also established a PPK model for this drug in critically ill patients (Garonzik et al., 2011). They found that creatinine clearance (CrCL) and body weight were key covariates influencing drug clearance and distribution volume, respectively. Based on Monte Carlo simulations, they modeled dosing regimens for patients receiving intermittent hemodialysis, continuous renal replacement therapy (CRRT), and those not undergoing dialysis, and developed the first dosing recommendations for CMS in critically ill patients undergoing extracorporeal renal therapies. These two studies provide important guidance for the individualized treatment of CMS. In 2010, Houk BE et al. used six clinical datasets on sunitinib and employed a meta-analysis combined with a PPK model to systematically quantify the relationship between sunitinib exposure and its efficacy/adverse reactions (Houk et al., 2010). The results showed that sunitinib exposure levels were closely associated with treatment outcomes. Higher drug exposure was found to extend time to tumor progression (TTP) and overall survival (OS), while the adverse reactions were mostly mild to moderate and manageable. Furthermore, in 2002 and 2000, Avramis VI et al. and Schuttler J et al., respectively using propofol and pegaspargase as representatives, employed PPK modeling methods combined with PK/PD analysis strategies, laying the theoretical foundation for the development of target-controlled infusion systems and the dose optimization of enzyme-based anticancer drugs (Schüttler and Ihmsen, 2000; Avramis et al., 2002). The studies mentioned above have provided standardized procedures in the application of NONMEM software, covariate modeling strategies, and combined pharmacokinetic simulations, which hold significant theoretical and practical value for subsequent individualized dosing research. These studies have greatly advanced the practical application of PPK models in clinical pharmacology and new drug development.

Over the past few decades, researchers have developed many PPK models for a wide range of populations with different physiopathological states. We utilized authors’ keywords co-occurrence analysis to help us identify populations and drugs that have received focused attention in this area. Our results indicate that special populations, such as children, critically ill patients, and those undergoing organ transplantation, seem to receive more attention from researchers. This is not surprising, as infants and children are in a phase of growth and development, with many organs not yet fully mature, and their physiological conditions and metabolic processes are continuously changing. As a result, pediatric dosing is more complex than adult dosing. Critically ill patients are characterized by the fact that their physiopathological state is quite different from that of ordinary patients, and is usually accompanied by multi-system involvement, multi-organ failure, and rapid changes in condition. Organ transplant patients, on the other hand, are characterized by large surgical trauma and susceptibility to infectious complications in the early postoperative period, and in the early post-transplantation period when organ function has not yet fully recovered, certain drugs metabolized by the liver and kidneys may be different from those of normal patients. Therefore, it is particularly important to establish a PPK model in these populations to assist in finding more rational and effective dosing in clinical practice.

In terms of specific drugs, the top 15 most frequently mentioned drugs are mainly focused on three categories: antimicrobial agents, immunosuppressants, and anticancer drugs. Among these, antimicrobial agents occupy six positions, indicating researchers’ special attention to antimicrobial drugs. Vancomycin and tacrolimus appeared significantly more frequently than other drugs and became the focus of researchers’ attention. Vancomycin is the first-line drug used in clinical practice to treat methicillin-resistant coagulase-negative staphylococci (MRCNS) and methicillin-resistant *Staphylococcus aureus* (MRSA). However, it has a narrow therapeutic index, may lead to specific nephrotoxicity and ototoxicity with prolonged or high dose use, and its pharmacokinetics show great individual variability (Rybak, 2006; Zhao et al., 2014). Tacrolimus, on the other hand, is now widely used as a drug for organ rejection after transplantation. However, it is characterized by poor oral bioavailability, narrow therapeutic window and large inter-patient pharmacokinetic differences, so it may lead to adverse reactions, such as nephrotoxicity, during treatment (McDiarmid et al., 1993; Jacobo-Cabral et al., 2015). In summary, we found that researchers preferred to focus on populations with rapidly changing disease and large inter-individual variability in PK parameters, as well as drugs with large pharmacokinetic differences and narrow therapeutic windows.

In addition, we identified current research hotspots in the field of PPK model. The results of the reference co-citation analysis and co-citation burst analysis indicate that researchers currently seem to focus more on establishing PPK models for antimicrobial drugs in critically ill patients and developing PPK models for tacrolimus in transplant patients. In addition, the use of external evaluation to assess the predictive performance of PPK models seems to be one of the recent focuses of researchers. In the overlay visualization map of authors’ keywords co-occurrence analysis, we found that “extracorporeal membrane oxygenation (ECMO)” is an emerging trend for future research. Critically ill patients often already have altered pharmacokinetics, and the use of ECMO systems may further affect the pharmacokinetic properties of patients. Specifically, ECMO can increase cardiac output and tissue perfusion thereby potentially altering the volume of distribution and clearance of drugs. The ECMO circuit also adsorbs drugs, especially those with lipophilic, low molecular weight and high protein binding properties, thereby decreasing drug plasma concentrations (Kriegl et al., 2024). However, current results on whether ECMO-assisted therapy affects the pharmacokinetics of drugs in patients are inconsistent, suggesting that more research is needed to address this issue in the future.

In addition, voriconazole and polymyxin b are also future trends in research. Voriconazole is a broad-spectrum triazole antifungal drug, which is commonly used in the clinic for the prevention or treatment of invasive fungal infection (IFI), but its therapeutic window is narrow, with large inter-individual and intra-individual variations, and a high incidence of adverse reactions, including hepatotoxicity and neurotoxicity (Ashbee et al., 2014). According to a review published by Shi CC et al., in 2019 (Shi et al., 2019), although inflammatory markers [e.g., C-reactive protein (CRP)], *CYP3A4*, the combination of various types of proton pump inhibitors and glucocorticoids, as well as various types of body weight markers [ideal body weight (IBW) and adjusted body weight (ABW)] have been demonstrated to affect voriconazole blood concentrations, only one study (Mangal et al., 2018) succeeded in including pantoprazole as a covariate in the model, and the rest of the above mentioned influences could not be included as covariates in the model for reasons that are not yet known (Mangal et al., 2018). Although three studies have successfully included CRP as a covariate in their models since the publication of this review, these studies have limitations (Jiang et al., 2022; Takahashi et al., 2022; van den Born et al., 2023). Therefore, future studies should consider trying to add the above influences as covariates to the model. Polymyxin b is commonly used in clinical practice as a last resort for the treatment of multidrug-resistant Gram-negative bacterial infections. Through a review published by Wang X et al., in 2024, it was noted that the covariates of PPK model on polymyxin b were mainly related to creatinine clearance, body weight, albumin, age, Sequential Organ Failure Assessment (SOFA) score, and continuous renal replacement therapy (CRRT), but results varied across studies, possibly due to reasons including small sample size, mostly single-center studies, and patient homogeneity (Wang et al., 2024b). We therefore expect that prospective, multicenter, large sample size studies will be conducted in the future to further clarify the effects of these covariates and optimize the clinical use of polymyxin b.

In recent years, the emerging concept of model-informed precision dosing (MIPD) has attracted a lot of attention from researchers. PPK model is regarded as a core tool for realizing MIPD. Currently, most implementations of MIPD are primarily based on the PPK model and patient-specific information (such as plasma concentration, previous medication records, and patient characteristics), and then use Bayesian methods to predict individual patients’ PK parameters and drug concentrations. Several studies have demonstrated the ability of the MIPD approach to improve the success rate of reaching therapeutic goals compared to dosing methods based on instructions guidance or based on standard therapeutic drug monitoring (TDM) (Abulfathi et al., 2018; Gastmans et al., 2022; Lu et al., 2022; Lloberas et al., 2023; Shi et al., 2023). Along with MIPD, external evaluation has also garnered attention, and the two are closely related. Simply put, external evaluation involves assessing the predictive performance of a model using a dataset independent of the one used to build the model. It is considered a very valuable and crucial step in determining whether a PPK model can be applied to clinical practice (Moons et al., 2012). In recent years, numerous studies have been devoted to the external validation of established PPK models (Broeker et al., 2019; Cheng et al., 2021; Corral Alaejos et al., 2022; Cheng et al., 2024). The central aim of external evaluation is to test the generalizability and extrapolation of the model across different populations, as well as to explore whether differences between different clinical settings may affect the broad applicability of the model. In addition, the difficulty of obtaining blood samples from certain specific populations limits modeling, and models that are externally validated with good extrapolation performance can save the time and cost required to build models in these populations. However, the results of most studies indicate that only a few models exhibit satisfactory predictive performance. This phenomenon may stem from the fact that models developed on specific populations are difficult to generalize to other populations due to pathophysiological differences, or due to the small size of patient samples used in constructing the models. The results of Broeker A et al. showed that models constructed based on large heterogeneous patient databases had the best predictive performance (Broeker et al., 2019). Utilizing large heterogeneous patient data may help models to better account for inter-individual differences. Therefore, we suggest that researchers should include large-scale patient population data as much as possible when constructing models in the future to enhance the generalization ability and prediction accuracy of the models. In addition, we have observed a growing interest among researchers in applying machine learning (ML) techniques to PPK models. The main advantage of ML is its ability to process and analyze large amounts of data and to mine the intrinsic features of the data. Several studies have now shown that ML methods based on PPK models are more accurate and stable in terms of model prediction performance than PPK model methods alone (Li G. et al., 2024; Tang et al., 2024; Wang Y. P. et al., 2024). Therefore, we suggest that researchers explore the method of combining PPK model with ML techniques in future studies, with a view to further improving the model’s prediction ability and application scope.

## 5 Limitation

Compared with traditional review articles, our study provides a comprehensive and quantitative analysis of research priorities, hotspots, and trends in the field of PPK model applications, an approach that helps scholars gain a deeper understanding of developments in the field. However, our study also has some limitations. First, due to the limitations of the analysis software, we only included publications from the WoS database. Second, the number of citations to a publication is affected by time, with earlier publications usually receiving higher citations, so some excellent research may not receive the recognition it deserves because of its relatively short publication time. Finally, our use of different literature analysis software may have resulted in missing information, which in turn affected the accuracy of the results. Despite these limitations, our study provides a comprehensive survey of hot topics and trends in the field of PPK modeling, providing valuable insights for current and future research.

## 6 Conclusion

This study provides the first comprehensive bibliometric analysis of PPK model-related publications between 2000 and 2024. Our findings reveal the rapid development of the PPK model worldwide and the growing interest of researchers in it. Through keyword co-occurrence analysis and reference co-citation analysis, we identified the research hotspots and emerging trends in the field, and the results showed that the research was mainly concentrated in the fields of antibacterial drugs, immunosuppressants and antitumor drugs. Patient populations for ECMO-assisted therapy, voriconazole, polymyxin b, external evaluations, machine learning and MIPD are emerging trends. Although PPK models offer new possibilities for optimizing therapeutic drug dosage and enabling individualized treatment, there is still some variation in the PPK models established in existing studies. We believe that models constructed using population data that are prospective, densely sampled, multicenter, and encompass large, heterogeneous patient populations may be more effective at revealing inter-individual differences, reducing model uncertainty, and enhancing the predictive power of the model to address the above issues. In addition, it has been shown that ML has a positive effect in enhancing the predictive power of PPK model, so we recommend the use of incorporating ML in future research. This study provides researchers, especially beginners, with insight into the applied research field of PPK model.
