# Supplementary material for: Global research on the utilization of population pharmacokinetic model: a bibliometric analysis from 2000 to 2024
Source: Front Pharmacol. 2025 May 12;16:1548023. doi: 10.3389/fphar.2025.1548023 (PMC12104660; doi:10.3389/fphar.2025.1548023)
Supplement: Supplementary file 1 [file Supplementaryfile1.docx]

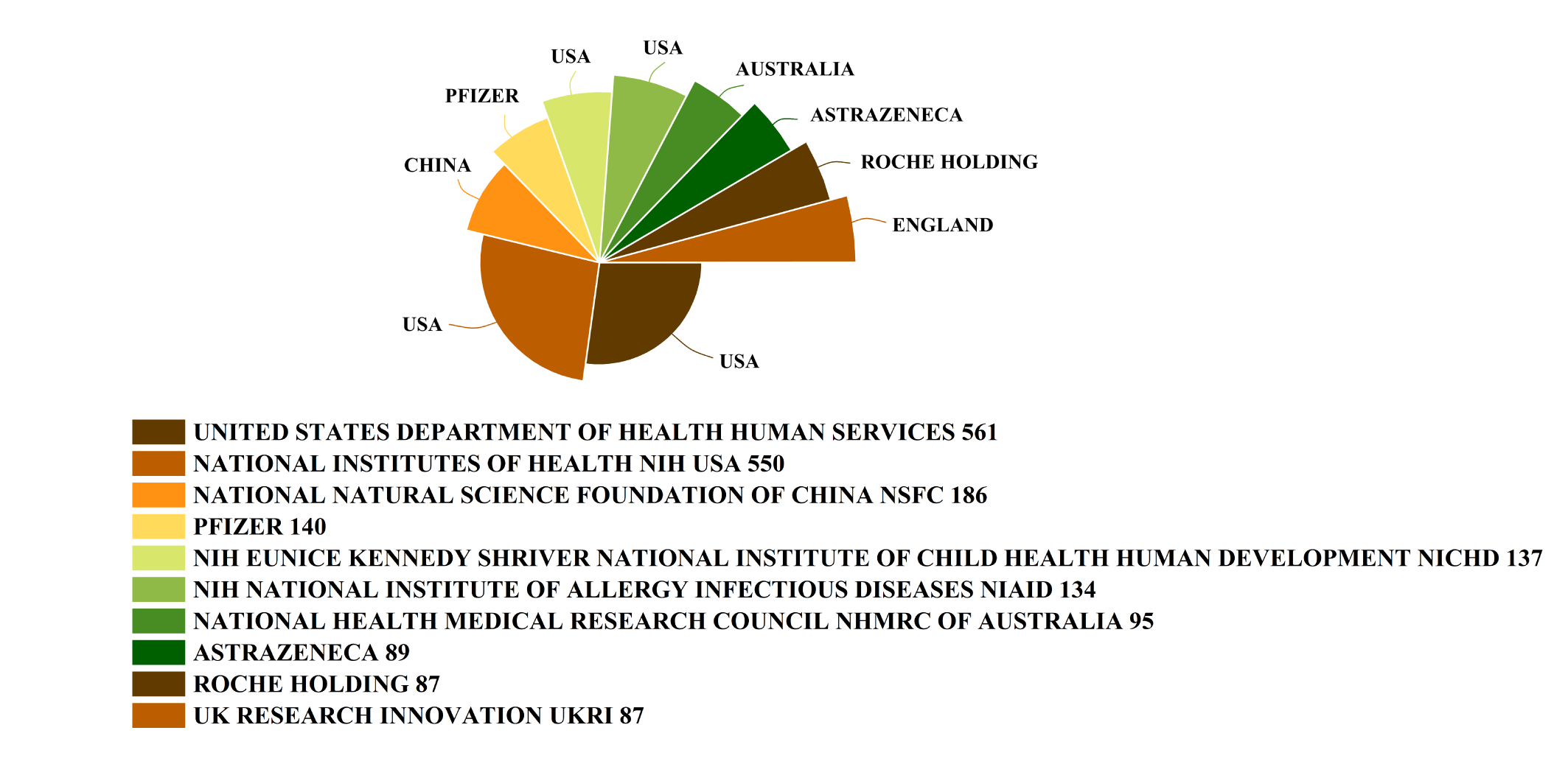


Supplementary Figure 1 Top 10 most funding agencies.


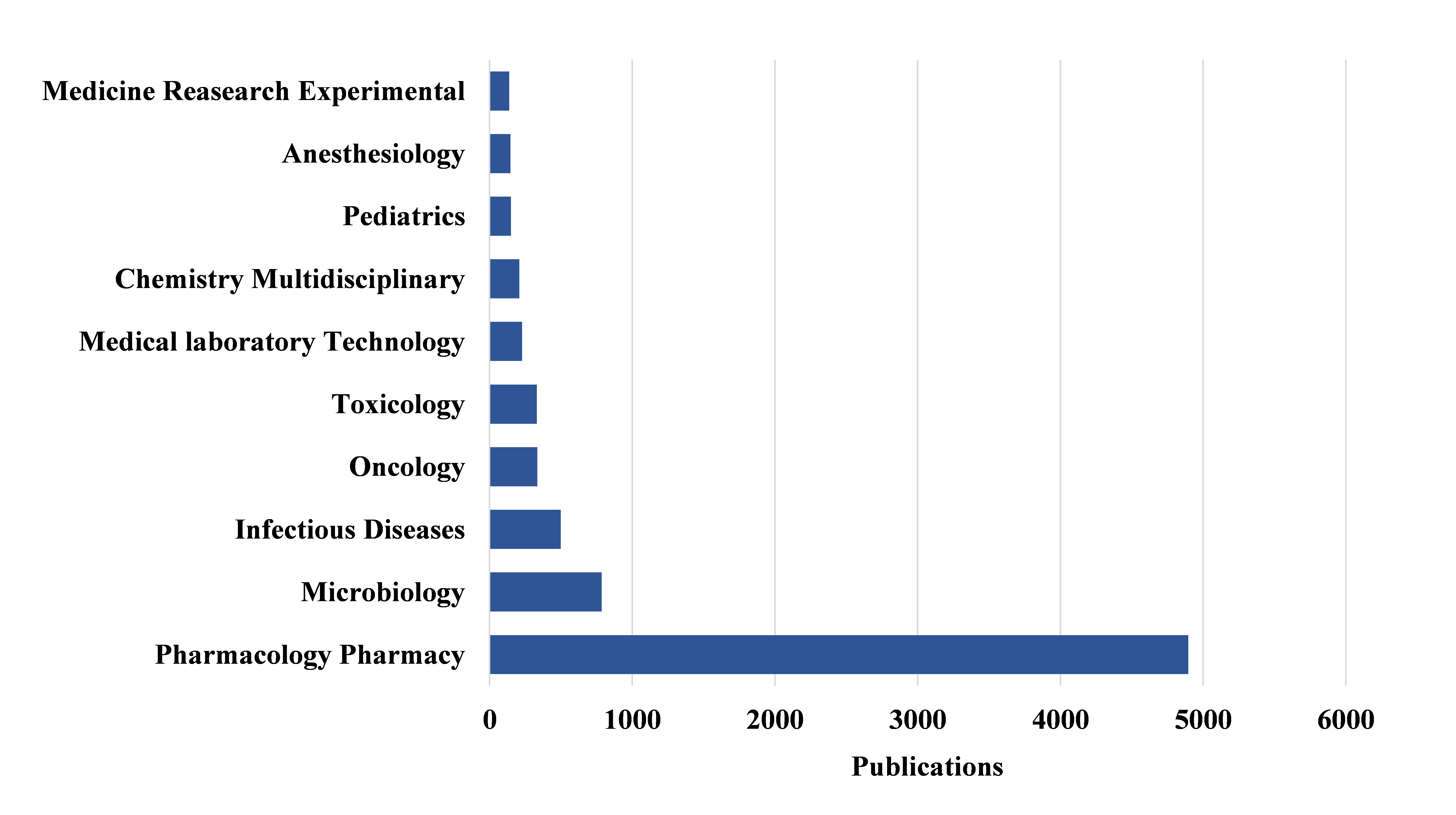


Supplementary Figure 2 Top 10 subject categories in terms of number of publications.
